# Brain Recovery after a Plane Crash: Treatment with Growth Hormone (GH) and Neurorehabilitation: A Case Report

**DOI:** 10.3390/ijms161226244

**Published:** 2015-12-21

**Authors:** Jesús Devesa, Gustavo Díaz-Getino, Pablo Rey, José García-Cancela, Iria Loures, Sonia Nogueiras, Alba Hurtado de Mendoza, Lucía Salgado, Mónica González, Tamara Pablos, Pablo Devesa

**Affiliations:** 1 Scientific Direction Medical Centre Foltra, Teo 15886, Spain; gussy_diaz@hotmail.com (G.D.-G.); preypa@gmail.com (P.R.); pepegarcia85@gmail.com (J.G.-C.); irilou_@hotmail.com (I.L.); sonianogueiras80@yahoo.es (S.N.); albitahmf@hotmail.com (A.H.M.); lucia89_9@hotmail.com (L.S.); mofrango@hotmail.com (M.G.); tamara_pisc@hotmail.com (T.P.); pdevesap@foltra.org (P.D.); 2 Department of Physiology, School of Medicine, University of Santiago de Compostela, Santiago de Compostela 15710, Spain

**Keywords:** GH, traumatic brain injury, neurorehabilitation, brain plasticity, adult neurogenesis, cognitive functions, spastic tetraplegia

## Abstract

The aim of this study is to describe the results obtained after growth hormone (GH) treatment and neurorehabilitation in a young man that suffered a very grave traumatic brain injury (TBI) after a plane crash. Methods: Fifteen months after the accident, the patient was treated with GH, 1 mg/day, at three-month intervals, followed by one-month resting, together with daily neurorehabilitation. Blood analysis at admission showed that no pituitary deficits existed. At admission, the patient presented: spastic tetraplegia, dysarthria, dysphagia, very severe cognitive deficits and joint deformities. Computerized tomography scanners (CT-Scans) revealed the practical loss of the right brain hemisphere and important injuries in the left one. Clinical and blood analysis assessments were performed every three months for three years. Feet surgery was needed because of irreducible equinovarus. Results: Clinical and kinesitherapy assessments revealed a prompt improvement in cognitive functions, dysarthria and dysphagia disappeared and three years later the patient was able to live a practically normal life, walking alone and coming back to his studies. No adverse effects were observed during and after GH administration. Conclusions: These results, together with previous results from our group, indicate that GH treatment is safe and effective for helping neurorehabilitation in TBI patients, once the acute phase is resolved, regardless of whether or not they have GH-deficiency (GHD).

## 1. Introduction

Classically, traumatic brain injury (TBI) has been considered “an alteration in brain function, or other evidence of brain pathology, caused by an external force” [1]. TBI is present in all societies as the most severe, disabling neurological disorder for millions of patients worldwide [2], but also a major cause of death, and economic cost in developed countries as well as the leading killer and disabler of young adults under the age of 35, since multiple functional and social impairments appear as a consequence of motor and cognitive affectations (lost of short-term memory, executive functioning, slow information processing speed, intelligibility of speech, altered behavior, *etc.*) [3].

A number of studies demonstrated that all neurological damage from TBI does not occur at the moment of impact, but evolves over the following hours and days; that is, a significant amount of central nervous system damage occurring after TBI resulted from secondary (diffuse axonal injury) brain injuries [1,2,4,5], which involves a complex cascade of biochemical events, usually leading to delayed tissue damage and cell death [6,7].

The damaged brain may recover part of its functions spontaneously, a process that may take several months or even years. However, early neurorehabilitation is needed to improve natural mechanisms leading to the recovery of the damaged brain and achieve the best possible functional and social functionality, considering the complexity of the sequelae (motor, cognitive, and emotional) caused in varying combinations by TBI. This partial recovery may occur as a consequence of two different physiological mechanisms that attempt to repair the damage produced by the brain injury: (1) quick proliferation of neural precursors; and (2) development of neural plasticity [8]. Both are independent but complementary of each other and both require the intervention of neurotrophic factors. Therefore, many attempts have been made for reinforcing the activity of these mechanisms and block the secondary cascade of events that increase the damage after TBI for minimize the injury suffered (for a detailed review see [9]).

Since the pioneer study of Chen *et al.* [10] in rats, a number of preclinical studies indicate that stem cell therapies are promising for treating TBI. Among them, direct intracerebral administration of mesenchymal stem cells (MSCs) have been reported to induce neuroprotective and regenerative effects following cerebral ischemia and TBI [11,12]. Currently, preclinical data show cell therapies to provide benefit when administered systemically or with transplantation to the site of injury. These studies have shown that these cells are able to attenuate the inflammatory response to injury and stimulate production of neurotrophic factors. In animal models, beneficial effects on blood–brain barrier permeability, neuroprotection and neural repair through enhanced axonal remodeling have been observed. However, clinical investigation with cell therapies for TBI remains ongoing [13].

Hence, apart of inducing prophylactic hypothermia for more than 48 h, which has provided unclear results in terms of patient outcomes [14], medical science has little to offer for acting acutely to avoid the persistent symptoms that prevent many of the individuals that suffer a TBI from fully re-entering society.

Therefore, for now, attempts for trying to recover TBI patients have to be restricted to begin once the acute phase of TBI has resolved and the patient is discharged from the hospital.

Over the last few years, several factors, with known neurotrophic activities, have been studied in order to reduce the sequelae caused by brain damage. The idea was that administering one of these factors (displaying neuroprotective effects, or acting on adult neurogenesis and/or brain plasticity) might facilitate the responses to adequate kinesitherapy thus improving the recovery. However, most of these factors did not prove to exhibit significant “curative properties” or they might produce important adverse side-effects [9].

GH is one of the factors that over the last few years has received considerable attention as a possible therapy directed towards repair of neural injuries, owing to its ability to promote neurogenesis in response to a brain damage [15].

The hypothesis that GH and insulin-like growth factor 1 (IGF-I) play a role on brain repair after an injury was postulated years ago [16,17,18,19], and a number of studies from our group and others demonstrated this, both in GH-deficient (GHD) [20,21,22,23,24,25,26,27,28] and non-GHD [8,29] patients with acquired brain injury.

**Figure 1:**
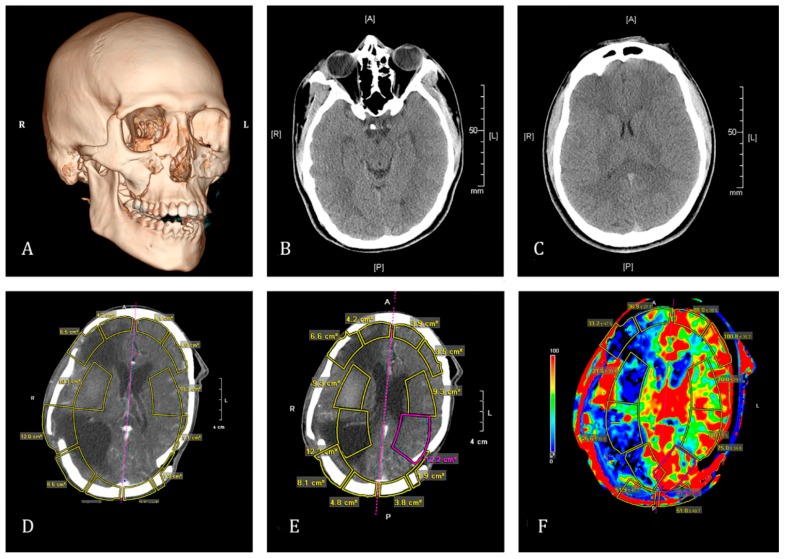
Immediate consequences of the TBI: (**A**) Brain CT-Scan performed four hours after the accident showing the fractures produced in the facial skeleton; (**B**,**C**) brain cerebral images from the same CT-Scan radiologically informed as normal; (**D**,**E**) images from a brain CT-Scan performed two months after the accident, observe the important destruction in the right hemisphere; and (**F**) corresponding image to the previous CT-Scan in (**D**,**E**) after administration of iodinated contrast. Notice the great lack of perfusion in the right hemisphere, while perfusion in the left one was informed as normal. CT- Scans: A = Anterior; P = Posterior; R = Right; L = Left.

This case report describes the results obtained in a non-GHD young man treated with GH and specific neurorehabilitation fifteen months after suffering a very severe TBI produced by a plane crash leading to a practical destruction of the right brain hemisphere (Figure 1), apart from severe polytraumatisms.

Our results show a practically complete recovery of the grave damage suffered, indicating that GH administration is safe and an effective help for kinesitherapy in non-GHD TBI patients, in accordance with our previous postulates [8,29].

## 2. Results

### 2.1. Physiotherapy

At admission, the Modified Ashworth scale score for spasticity was 4 for the left hemibody and 3 for the right one (normal value = 0; range = 0–4), while the Penn scale for spasm frequency was 3 (more than one spasm per hour; normal value = 0; range = 0–4). Functional Ambulation Classification (FAC scale; normal value = 5; range = 0–5) was 0. Trunk Impairment Scale (TIS; normal value = 23; range = 0–23) was 5.

These values were progressively improving during the treatment until reaching normal values in Penn, TIS and the Modified Ashworth scale.

**Figure 2:**
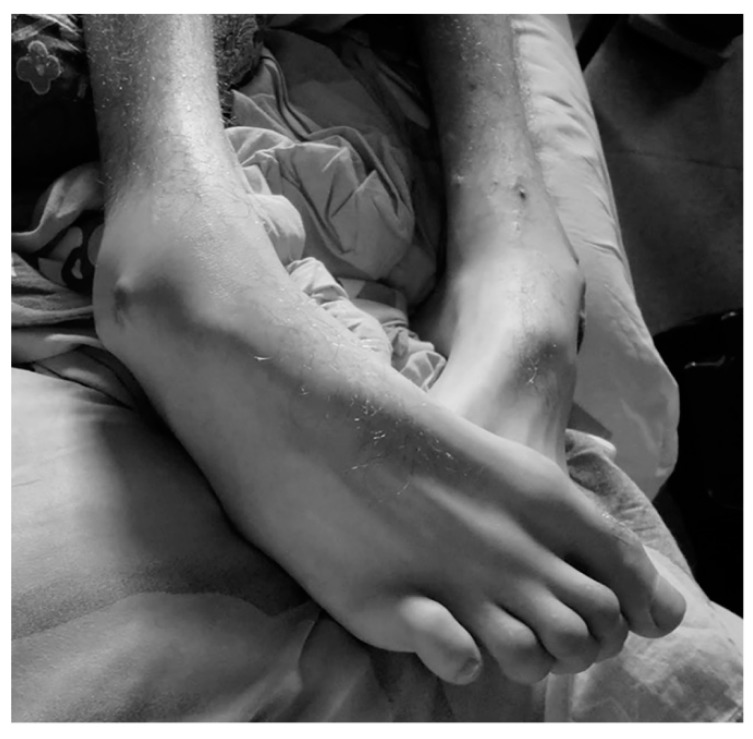
Irreductible equinovarus feet produced by the strong spasticity that the patient developed after suffering brain trauma.

**Figure 3:**
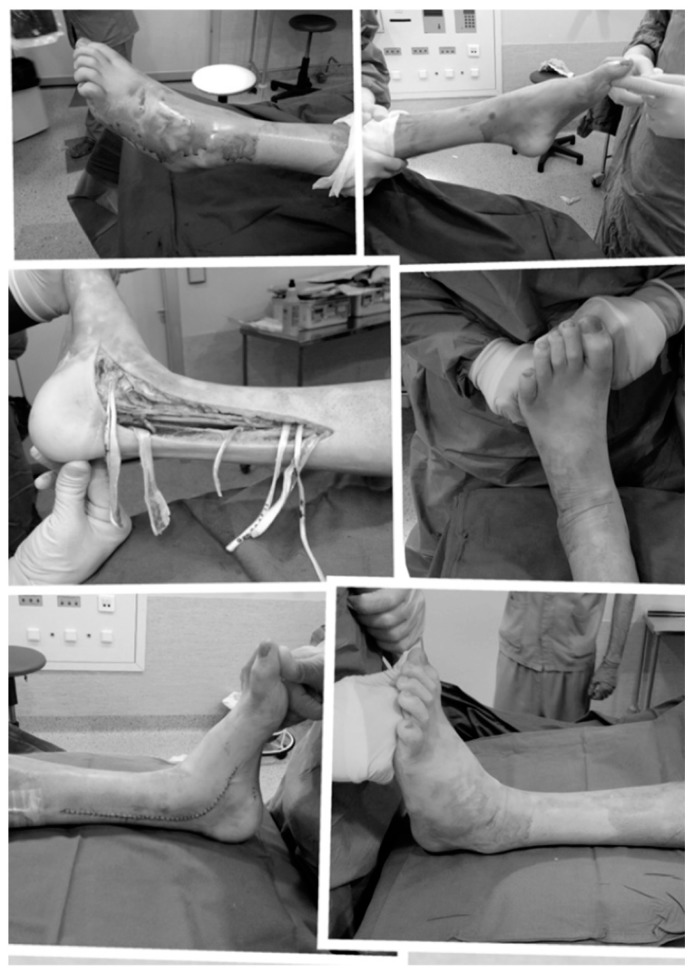
Feet surgery: (**Upper images**) Equinovarus feet before the surgery; (**Middle**) Left, surgery consisted in legs tendons transpositions; and right, the surgeon is testing the correction of equinovarus after the surgery; (**Down**) Equinovarus has been corrected in both feet.

Given the advances observed in this area, fifteen months after beginning the treatment, the patient was able to correctly and voluntarily extend and flex his left leg when lying on a bed, a corrective surgery of his equinovarus feet (shown in Figure 2) was scheduled (Figure 3).

Eight months after the surgery was performed, training for walking began, both with the therapists and in the LokoStation, and later in the swimming pool.

**Figure 4:**
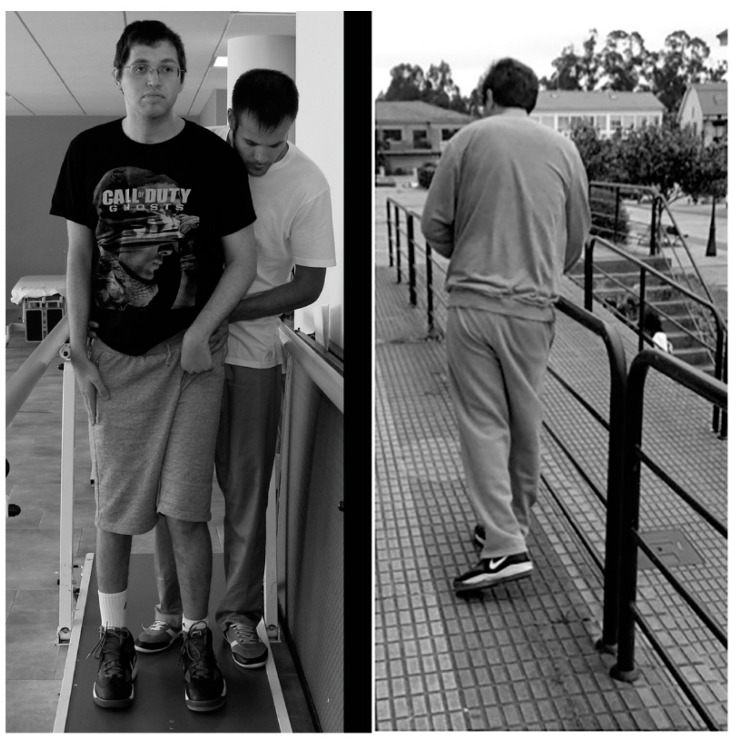
Working and walking after the feet surgery: (**Left**) the patient beginning to walk in parallel bars with the help of a physiotherapist and (**Right**) the patient beginning to walk alone on the street. Observe the movement of his left foot and leg.

**Figure 5:**
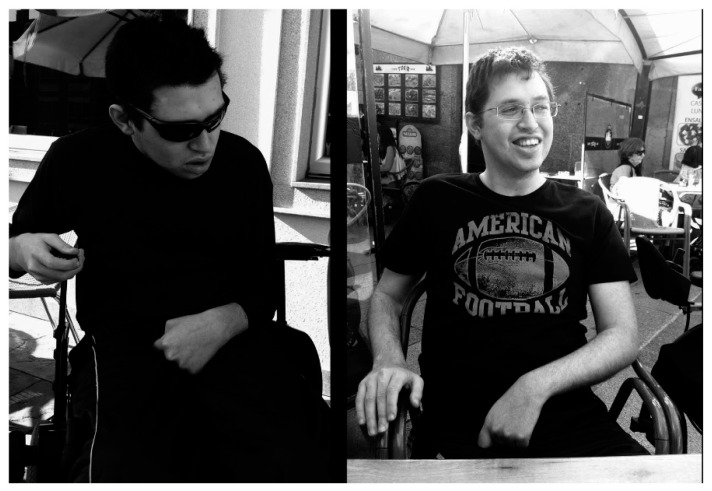
The changes: (**Left**) The patient two months after admission. Notice the weak control of head and trunk, the patient sitting on a wheel chair, the right and left arms flex and the irreducible carpal flex of the left wrist; (**Right**) The patient three and a half years later. Observe the patient sitting on a normal chair, the normal position of head, trunk and both arms, the corrected flex of both arms and the almost corrected flex of the left hand.

Thirty-four months after commencing the treatment, the score reached in the scale of FAC was almost normal (4 over 5); Figure 4 shows how the patient began to be able to walk in parallel bars with the help of a therapist, and then alone about 300 m, but with a support, because of the irreducible flexion of the right wrist that made him afraid of falling. Tinetti scale for measuring gait and equilibrium reached a value of 16 (normal = 28; range = 0–28) at this time, while being impossible to be measured at admission. Six months later, the patient walked alone and returned to his normal life (Figure 5).

### 2.2. Psychomotor Stimulation

The Wechsler adult intelligence scale (WAISS III test), indicative of the cognitive abilities of the patient, could not be performed at admission due to the intellectual deficits that the patient presented. However, cognition was improving after the first three months of treatment, and ten months later, his IQ score was 71, while at the end of the treatment the test indicated an IQ score of 95, a value slightly higher than mean values in the normal population. That is, the patient recovered perceptive organization, processing speed, verbal comprehension, working memory and operative memory. This was confirmed by the results obtained in another test, MMSE (Mini Mental State Examination), that evolved from a value of 1 at admission to 27 at the end of the treatment period (range = 0–30), indicating that spatiotemporal orientation; attention, concentration and memory; abstraction capacity; language ability; and visuospatial perception and the ability to follow commands clearly improved and no significant cognitive deficits existed.

### 2.3. Speech Therapy

At admission, the test measuring the Intelligibility of Speech had a value of 2 points (normal value: 5 points; range = 0–5), while two years later it increased to 4 points. The House–Brackmann scale for facial paralysis decreased from 3 to 1 points (normal value = 1; range = 1–6), and the FOAMS scale for deglutition increased from 2 to 7 points (normal = 7; range = 1–7) one year after rehabilitation commenced. According to these results, tracheostomy was closed and the gastric tube was removed.

### 2.4. Occupational Therapy

The Barthel Index measuring daily life activities increased from 0 points (maximal dependence) at admission to 70 points at the end of treatment period (normal value: 100; range = 0–100). The left carpal structured flexion impeded him to obtain a higher value in this index. Similarly, the Loewenstein Occupational Therapy Cognitive Assessment (LOTCA) battery progressively increased from 34 points obtained when the test was first performed (one year after commencing the treatment) to 73 at the end of the treatment (range = 21–87).

### 2.5. CT-Scan

**Figure 6:**
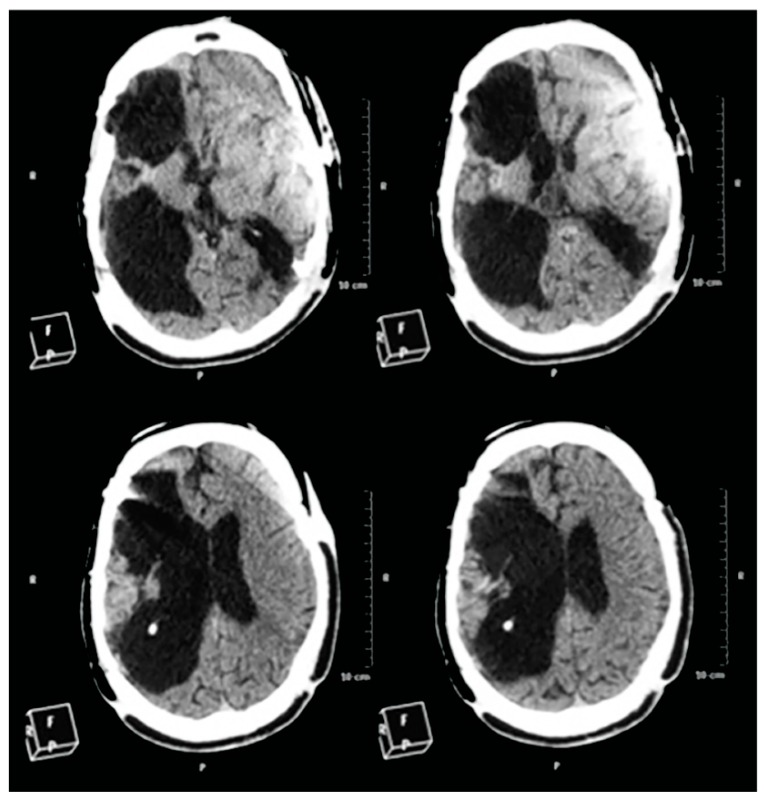
Two years of treatment. Despite the clinical improvements, a new CT-Scan revealed that the brain injury was higher than that observed in the first days after the accident and in the CT-Scan carried out two months later. Injuries in the right hemisphere were increased and new injuries appeared on the left one. Scale bar: 12 cm. F: Front. R: Right: P: Posterior.

Two years after commencing the treatment, a new brain CT-Scan was performed. Contrary to expectations, according to the evolution of the patient, the images obtained (Figure 6) revealed an extensive porencephalic lesion in the right brain hemisphere with a severe “*ex vacuo*” dilatation of the right lateral ventricle and moderated enlargement of the left lateral ventricle and a porencephalic lesion at the left parieto-occipital cortex. The radiologist also indicated that there was brainstem atrophia, related to wallerian degeneration, and cerebellar atrophia. With regard to the images observed in Figure 1, it is likely that these new brain damages occurred as a consequence of the axonal diffuse injury during the time that the patient remained in coma and/or the new craniectomies were carried out.

### 2.6. Blood Analysis

Routine blood analyses (hematimetry and biochemistry) were normal during all the treatment period. Plasma IGF-I values ranged between 331 and 559 ng/mL, regardless of whether or not GH treatment existed or there was a resting period. Plasma TSH and fT4 were in normal values during the whole treatment period. Similarly, plasma values of cortisol, testosterone, prolactin, IGFBP3 and tumoral markers (Prostate Specific Antigen (PSA) and Carcino Embryogenic Antigen (CEA)) fluctuated in normal values along the whole treatment period and at discharge.

GH administration did not induce any kind of adverse effects.

## 3. Discussion

As described in the Introduction, stem cell therapies are promising for treating TBI during the acute phase of the injury [11], because of attenuating the inflammatory response to injury and stimulating the production of neurotrophic factors. However, that study was carried out in preclinical models and it seems that the effectiveness of these implanted MSCs is limited to a therapeutic time window up to 72 h after the injury (more recently, seven days in human patients having suffered an ischemic stroke [30]), decreasing their effectiveness after that time, most likely because of the death of these cells. Perhaps, if the implant were accompanied by the administration of a survival factor, such as GH, the limited time window would increase.

Trophic factor secretion is postulated as a primary or secondary mechanism of action for these transplanted cells, however, there is little evidence to support trophic production by transplanted cells *in situ*.

In any case, both the limited time window for stem cells implants and the need for an adequate technology, not easily found in most hospitals, are limiting factors for minimizing the injuries suffered during the acute phase after a TBI.

While current evidence indicates that the adult brain can be repaired and regenerated after TBI, and both processes can be enhanced by pharmacological treatment, there remain significant gaps in knowledge and there is paucity of therapies to limit the disabling consequences of TBI or to promote neuroregeneration [31], despite the fact that a number of studies have been seeking pharmacological therapies targeting for increasing angiogenesis, axon guidance and remodeling, remyelination, neurogenesis and synaptogenesis, or for increasing the ability of pluripotent cells to differentiate into neurons, glia or vascular endothelium (for a detailed review see [31]).

Hypopituitarism is a frequent consequence of TBI [32,33,34] and a major cause of treatable morbidity among TBI survivors [33]. Among pituitary deficits after TBI, isolated GHD is the main finding observed reaching a prevalence significantly higher than other pituitary hormonal deficits [35]. However, and despite his important brain damage, the patient we treated did not have any pituitary hormonal deficits.

A number of data support a role for GH itself in neurogenesis and brain plasticity [15,16,17,18,20,21,22,23,24,25,26,27,36,37,38], two mechanisms responsible for brain repair after an injury. However, most of these studies have been carried out in rats or in GHD patients.

Exogenously applied GH promotes the proliferation and migration of neural stem cells derived from fetal human forebrains [39], and the proliferation of hippocampal neural precursors after brain injury induced by kainate administration, suggesting that GH treatment may cooperate with hippocampal GH in the repair of neurotoxic damage [15]; similarly, peripheral GH administration increases the generation of new brain cells in a number of cerebral areas in normal adult female rats [40]. According to this evidence, it is likely that GH may facilitate the proliferation, differentiation and survival of new neurons in response to brain injury. To date, only few studies in humans explore such a possibility indicating a positive effect for GH treatment together with specific neurorehabilitation [20,21,22,23,24,25,26,27,28]; but all patients in these studies had GH deficiency, and only one study [8] describes that GH administration, together with rehabilitation, may be useful for the recovery of TBI patients without GHD.

However, given the preclinical evidence and since GH induces the expression and/or the release of a number of factors with known neurotrophic properties (for a detailed review see [8]), it seems logical to assume that the hormone may also facilitate the response to rehabilitation therapies in TBI patients without GHD, as it was the case of the patient we treated and describe here. At this point, it is difficult to clearly delimitate the contribution of GH treatment or neurorehabilitation to the improvements observed. However, our previous experience in some non-GHD patients that refused to be treated with the hormone and only received neurorehabilitation indicates that the hormone plays a key role in the recovery. The improvements observed in patients receiving only neurorehabilitation were in general of poor magnitude and appeared after quite longer periods of rehabilitation (unpublished data). The same was observed in rats after a severe brain injury. Animals treated with GH and rehabilitation soon achieved a full recovery of their injury, while no improvements were observed in rats treated with vehicle and rehabilitation [36]. Therefore, it is feasible to conclude that GH administration provides a kind of support that makes rehabilitation much more effective. This is regardless of whether the patients suffer from GHD or not, although in GHD patients positive responses appear earlier, mainly at the cognitive level [20,21,24].

The fact that, in the patient here studied, cognitive improvements improved earlier and were more important than motor improvements agrees with emerging data indicating that GH treatment has an important role in improving cognitive function [41]; this seems to be important in our case study because pituitary GH secretion was normal in the patient we treated.

Of interest too in this case is the fact that plasma values of IGF-I fluctuated in a normal range during the entire treatment, regardless of whether or not the patient received GH treatment.

In summary, we describe a case of a young patient that, after having suffered a very important TBI, recovered a practically complete normal life after being treated with GH and specific neurorehabilitation.

Since the last brain imaging study did not reveal positive changes with regard to the previous imaging studies carried out in the patient, we think that the role played of GH in this case was on brain plasticity, at the expense of creating new neurons and synapses able to substitute those lost after the accident and surgical interventions [36], and, perhaps, to affect excitatory circuits involved in synaptic plasticity. The long-term treatment period of neurorehabilitation was mainly determined by the important structural abnormalities (joints, hips, spine, and feet) that the patient presented at admission.

According to these data and previous data from our group [20,21,24,25] and others [22,23,26,27], we suggest that GH treatment might be a safe and effective pharmacological therapy to help neurorehabilitation in TBI patients, regardless of whether or not they have GHD.

## 4. Experimental Section

The patient was an 18-year-old male that had a plane accident, suffered when the engine failed after takeoff, crashing into the ground from an altitude of 100 m. After the crash, he remained unconscious for an undetermined time.

### According to the Medical Files Provided by the Family of the Patient

At admission in his hospital of reference, the patient was conscious, reactive (Glasgow Coma Scale (GCS) score 15), isochoric and photoreactive pupils. The brain and cervical CT-Scan performed did not evidence any brain injury or any area of cerebral bleeding. The diagnosis was severe polytraumatism: fracture of the facial skeleton, zygomatic arch and malar bone, comminuted fracture of right tibia and fibula, fracture of the right astragalus and mobility of the whole dental arch corresponding to the upper jaw. The patient was sedated and admitted to the Intensive Care Unit (CU) to wait for reconstructive surgery scheduled for two days later.

Fourteen hours later, and due to the worsening of the patient, the family decided to move him to another hospital in the area. The incoming report describes a patient in very bad condition, breathing assisted with mechanical ventilation and GCS score 6. One day later, a new cerebral CT-SCAN evidenced a very important brain edema. Since intracranial pressures (ICPs) were uncontrolled, the patient underwent a right decompressive hemicraniectomy performed the next day. After this, ICPs continued to be constantly high, so a new cranial surgery was carried out three days later. A brain MRI study performed some days after the second cranial surgery indicated that the corpus callosum had decreased volume and that a number of microbleedings could be seen in sequences T2 (data not shown). This indicated the existence of a diffuse axonal injury.

**Figure 7:**
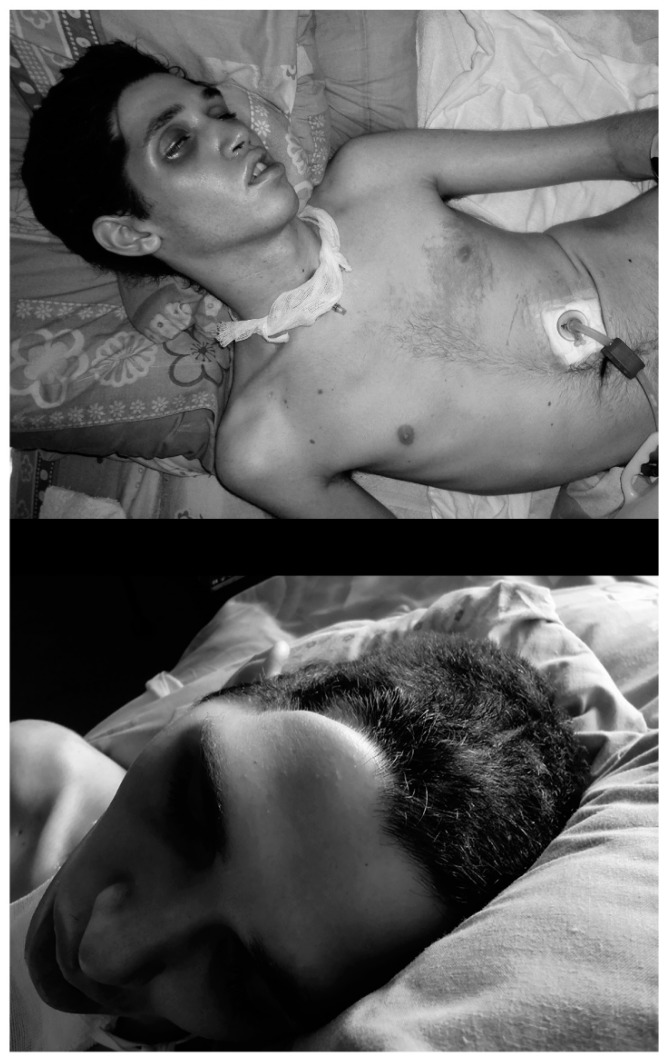
Deep coma occurred as a consequence of the axonal diffuse injury. (**Upper**) Few days after the accident occurred, the strong spasticity, more prominent in the left hemibody, led to the apparition of dorsal-lumbar scoliosis. Notice the gastric tube for feeding and the tracheostomy; (**Lower**) Remarkable parietal bones collapse.

A new CT-Scan performed two months after the accident, before and after administration of iodinated contrast, showed the existence of important encephalomalacia in the right brain hemisphere, subdural hematoma and subungueal emphysema, while no anomalies in the perfusion were observed in the left hemisphere and in the subtentorial nervous tissue (Figure 1). Three new craniectomies were performed in the following six months because the parietal bones, previously removed to decompress the cranial cavity, had collapsed into the skull (Figure 7) producing an increased cerebral pressure. This happened two more times at the left parietal side.

The patient remained in deep coma for nine months (Figure 7).

As Figure 7 shows, the patient had to be fed by gastric tube, and a tracheostomy allowed aspiring bronchial secretions. Daily rehabilitation attempted to avoid joint deformities and to wake up the patient from the coma. Six months after waking from coma, he was admitted to our medical center, fifteen months after the accident occurred.

At admission to Medical Center Foltra, the patient was fully dependent for daily life activities. He presented spastic tetraplegia, more prominent in the left hemibody, with upper members in flexion and lower members in extension. Right wrist and fingers were in irreducible flexion: Babinsky + bilaterally. There was left heminegligency; right hemianopsia; photoreactive pupils, but tending to mydriasis; complete loss of spatiotemporal orientation; dysarthria and very poor language; loss of short-term memory, low processing speed and attention deficit; lack of cephalic and trunk control; moderate cervico-dorsal kyphosis and dorsal-lumbar scoliosis (Figure 7); irreducible equinovarus feet (Figure 2); and lack of sphincter control.

The patient still maintained the tracheostoma, and had to be fed by gastric tube.

An electrophysiological study carried out by stimulation of the posterior tibial nerve at the ankle indicated an altered bilateral conduction in the somatosensorial pathway, showing loss of functional nerve units (data not shown). On the other hand, the analysis of visual evoked potentials carried out by monocular stimulation with flash indicated an abnormal morphology and increased latency of the conduction from both eyes (data not shown).

Blood analyses were normal and no pituitary deficits existed according to endocrine studies performed at admission, 15 months after the accident occurred. Values found were: Thyroid Stimulating Hormone (TSH) 2.48 µU/mL; free Thyroxine (fT4): 1.38 ng/dL; cortisol (8 am: 22 µg/dL, and at 8 pm: 8.4 µg/dL); testosterone: 6.6 ng/mL; and prolactin: 8.5 ng/mL. GH peak in response to clonidine test was 12 ng/mL. Insulin Gowth Factor-I (IGF-I) value was 559 ng/mL (range for the age of the patient: 127–585 ng/mL) and Insulin Growth Factor Binding Protein (IGFBP3): 7.1 µg/mL (range for the age: 3.1–7.3 µg/mL). We also measured plasma PSA (0.15 ng/mL) and CEA (2.4 ng/mL).

Studies and treatments were conducted according to the protocols of Medical Center Foltra in compliance with national legislation and the Code of Ethics of the World Medical Association (Declaration of Helsinki). After obtaining signed informed consent of their legal representatives, the patient was scheduled for GH treatment and rehabilitation consisting of daily physical therapy, speech therapy, psychomotor stimulation, occupational therapy (1 h/day for all of them), and visual stimulation with a tachistoscope (2 sessions of 15 min/day).

Once the conditions of the patient improved, in addition to the above-described therapies, he was scheduled for robotic gait trainer (LokoStation, fysiomed, Prague, Czech Republik; 3 days/week, 20 min per session) and therapy in swimming pool (3 days/week, 45 min per session). Clinical assessments and blood analysis (including thyroid hormones, cortisol, prolactin, testosterone, IGF-I and IGFBP3, and tumoral markers PSA and CEA) were performed every three months during the entire treatment.

GH treatment (Nutropin, Ipsen) consisted of one dose of 1 mg/day (subcutaneously), five days per week, for three months, followed by one month without treatment; after one month, GH was administered again at the same dose. This protocol procedure was continued for one year. Then, after a three-month resting period, the GH treatment protocol before described was repeated. Total GH administration lasted two years and three months. During resting periods, rehabilitation continued as scheduled, excepting for physiotherapy of legs that had to be discontinued for eight months due to surgery of the feet. GH treatment was always administered in the morning some minutes before commencing a session of physiotherapy or physical exercise. The reason for this was to avoid slight increases of glycemia, since GH is a counterregulatory hormone that opposes the effects of insulin.

Three months after initiating the rehabilitation, all the analgesics, anti-inflammatories and muscle relaxants were paulatinally retired.

In total, the neurorehabilitation treatment lasted three and a half years. Even though it is well known that pituitary function may worsen over time in TBI patients, final blood analyses before discharge indicated that no pituitary dysfunctions existed; in the case of GH, a new clonidine test revealed that GH response continued to be normal (GH peak = 15 ng/mL). The patient gave us a signed informed consent to publish his pictures.

## 5. Conclusions

GH treatment seems to be an important help for neurorehabilitation in TBI patients, regardless of whether they have GH-deficiency or not. The mechanisms of GH positive effects on brain recovery are not well known but they may include: (1) neural stem cells proliferation, differentiation and migration; (2) increasing synaptic circuits; (3) increasing blood supply to the brain; and (4) increasing the brain turnover of catecholamines: Noradrenaline (NA) and Dopamine (DA). All of these mechanisms, directly or indirectly mediated by GH, have already been described as involved in GH actions at the brain level.
